# A de novo assembled high-quality chromosome-scale *Trifolium pratense* genome and fine-scale phylogenetic analysis

**DOI:** 10.1186/s12870-022-03707-5

**Published:** 2022-07-11

**Authors:** Zhenfei Yan, Lijun Sang, Yue Ma, Yong He, Juan Sun, Lichao Ma, Shuo Li, Fuhong Miao, Zixin Zhang, Jianwei Huang, Zengyu Wang, Guofeng Yang

**Affiliations:** 1 College of Grassland Science, Qingdao Agricultural University, Qingdao, 266109 China; 2 Key Laboratory of National Forestry and Grassland Administration on Grassland Resources and Ecology in the Yellow River Delta, Qingdao, 266109 China; 3 Berry Genomics Corporation, Beijing, China

**Keywords:** *Trifolium pretense*, Genome, *De novo* assembly, PacBio HiFi, Genome annotation

## Abstract

**Background:**

Red clover (*Trifolium pratense L*.) is a diploid perennial temperate legume with 14 chromosomes (2n = 14) native to Europe and West Asia, with high nutritional and economic value. It is a very important forage grass and is widely grown in marine climates, such as the United States and Sweden. Genetic research and molecular breeding are limited by the lack of high-quality reference genomes. In this study, we used Illumina, PacBio HiFi, and Hi-C to obtain a high-quality chromosome-scale red clover genome and used genome annotation results to analyze evolutionary relationships among related species.

**Results:**

The red clover genome obtained by PacBio HiFi assembly sequencing was 423 M. The assembly quality was the highest among legume genome assemblies published to date. The contig N50 was 13 Mb, scaffold N50 was 55 Mb, and BUSCO completeness was 97.9%, accounting for 92.8% of the predicted genome. Genome annotation revealed 44,588 gene models with high confidence and 52.81% repetitive elements in red clover genome. Based on a comparison of genome annotation results, red clover was closely related to *Trifolium medium* and distantly related to *Glycine max, Vigna radiata, Medicago truncatula*, and *Cicer arietinum* among legumes. Analyses of gene family expansions and contractions and forward gene selection revealed gene families and genes related to environmental stress resistance and energy metabolism.

**Conclusions:**

We report a high-quality de novo genome assembly for the red clover at the chromosome level, with a substantial improvement in assembly quality over those of previously published red clover genomes. These annotated gene models can provide an important resource for molecular genetic breeding and legume evolution studies. Furthermore, we analyzed the evolutionary relationships among red clover and closely related species, providing a basis for evolutionary studies of clover leaf and legumes, genomics analyses of forage grass, the improvement of agronomic traits.

**Supplementary Information:**

The online version contains supplementary material available at 10.1186/s12870-022-03707-5.

## Background

**Fig. 1:**
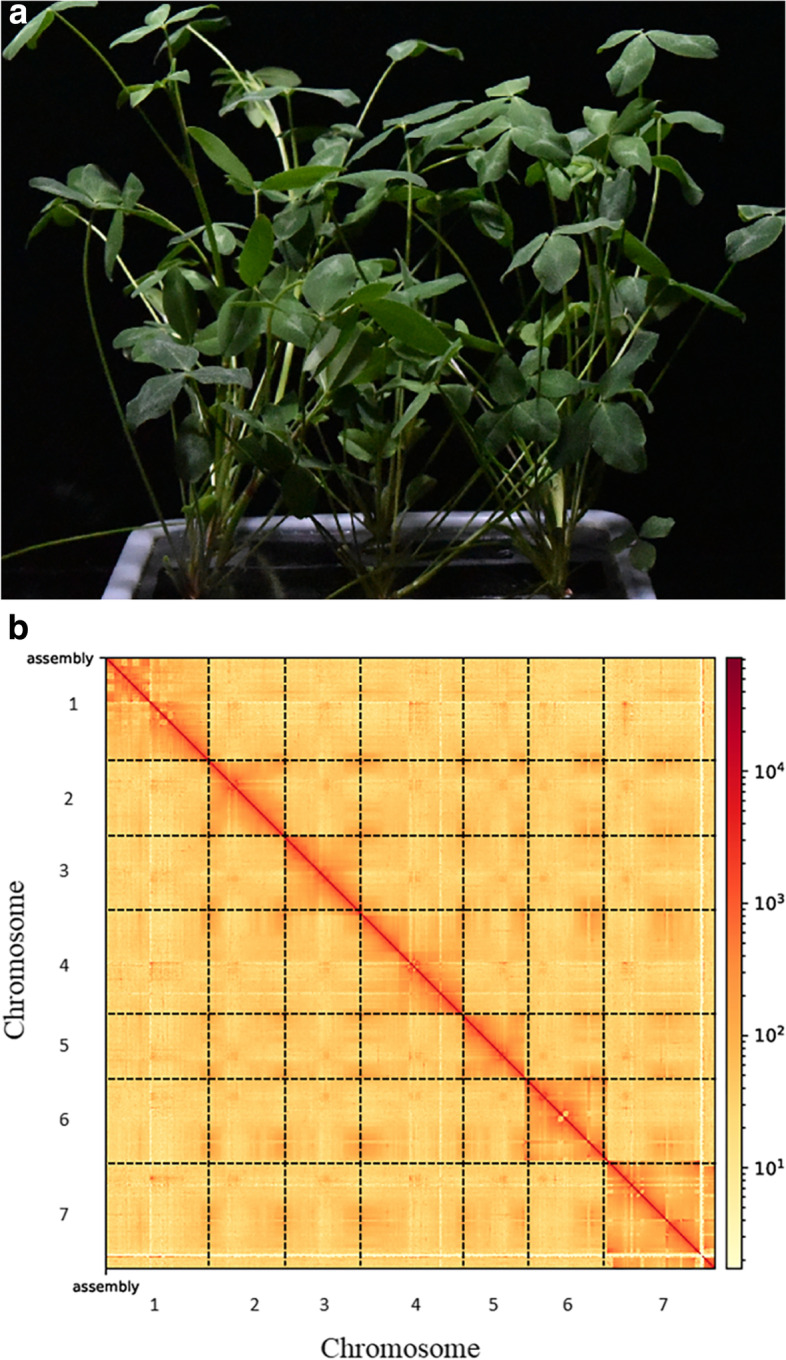
Plant morphology and Hi-C-assisted genome assembly of red clover. **a** Phenotype of the sequenced red clover plant. **b** Hi-C interaction heatmap showing 100-kb resolution super scaffolds

Red clover (*Trifolium pratense* L.) (Fig. 1a) is a temperate legume (*Leguminosae*) native to Europe and West Asia. It is not only inexpensive and rich in nutritional value but also contains a crude protein content of about 20%, with dry matter digestibility of about 70%, making it highly palatable and easily eaten by animals [1, 2]. Like alfalfa, red clover is self-incompatible and can be crossed with other subspecies [3, 4]. Its rich genetic diversity enables the production of many excellent agronomic traits. Red clover not only serves as a high-quality livestock feed but also has environmental and medicinal value [5–8]. As an important feed for dairy and beef cattle, red clover has important implications for the dairy and beef industries [9–11]. Although the world is affected by the new crown rot epidemic, the number of beef and dairy cattle is increasing, with corresponding increases in demand for high-quality forage [4, 12]. Red clover also has implications for environmental protection as well as medicinal value. It can co-produce nitrogen with *Rhizobium*, reducing the need for nitrogen fertilizers [13, 14]. Its developed root system promotes the reproduction of soil microorganisms and the growth of surrounding trees [7, 15]. Red clover contains high amounts of anthocyanins and is a potential candidate for reducing inflammation and oxidative stress [16]. Despite these advantages of red clover, its disadvantages include flatulence in ruminants, cold tolerance, and root sensitivity to soil pH. Accordingly, the development of improved varieties by plant breeding is an important goal.

As a non-model legume, the structure of the red clover genome and important genetic information have not been fully determined, substantially limiting breeding and crop improvement. Relative to closely related species (e.g., white clover, alfalfa, and soybean) [17–19], progress in genomic research on red clover has been slow. The genome structure was initially explored by fluorescence in situ hybridization [20, 21]. Subsequently, simple repeat sequence (SSR) and single nucleotide polymorphism (SNP) markers were used to construct a red clover linkage genetic map [22]; however, few genes were mined, and the impact on variety improvement was limited. Subsequently, the red clover genome was constructed using the Illumina HiSeq 2000 platform; however, the final assembly results were insufficient for data mining in terms of continuity and completeness [23]. Therefore, it is necessary to construct a high-quality red clover genome to accelerate genetic and genomic research on red clover and future breeding.

In this study, we obtained a high-quality chromosome-level red clover genome by using Illumina, PacBio HiFi, and Hi-C data. The assembly contained 96 scaffolds (~ 423 Mb), with N50 = 55 Mb, accounting for 92.8% of the estimated genome size (456 Mb). Compared with that of the previously reported red clover genome assembly [23], the quality was greatly improved. From the genome, we annotated 44,588 genes. Furthermore, we evaluated gene family expansion and contraction and positive selection and performed phylogenetic analyses. In addition, analyses of the collinear relationship between the chromosomes of red clover and closely related species supported the accuracy and sensitivity of the data for studies of genomic evolution and for predictions of genomic patterns [24, 25]. Complete genomic information for the species and closely related species provides a basis for studying the differentiation and evolution of the species [26, 27]. This study provides a new starting point for evolutionary genomics research and a new research direction for analyzing the evolutionary relationships between red clover and related species.

## Results

Genome-survey, sequencing, and assembly. The size, duplication rate, heterozygosity, and other parameters of the red clover genome were assessed by Jellyfish [28]. After quality control, Illumina sequencing yielded 26 Gb of data. We randomly selected 10,000 clean reads for comparison with the NT, and the rate of successful alignment against plant genomes (*Medicago truncatula, Trifolium repens, Cicer arietinum and Trifolium meduseum*) was 98.63%. A K-mer analysis further predicted a genome size of 456 Mb, with heterozygosity accounting for 1.57% and repeat sequences accounting for 54% of the genome.

We used traditional next-generation sequencing (NGS) data assembly methods to predict the genome size and third-generation HiFi sequencing (TGS) developed by PacBio for assembly of the red clover genome. In addition to making up for shortcomings of NGS in assembly applications, TGS does not require PCR amplification, produces ultra-long reads, GC preference and genome assembly by directly using high quality HiFi reads for splicing, which simplifies the process of sequencing and assembly, and improves the accuracy of assembly results [29–31]. High-quality HiFi reads were obtained after correcting subreads for ccs processing. In total, 1,764,862 HiFi reads was generated, and the N50 length was 18 kbp.

Contigs were then generated according to the phased string graph [32]. The assembled genome (423 Mb) contained 194 contigs, with an N50 size of 13 Mbp, and the largest contig was 34 Mbp. The average GC content of the assembled genome was 33.6%, which was slightly lower than those of the previously reported genomes of *White lupin* (33.7%) [33] and *Pisum sativum* (37.6%) [34]. The Illumina reads were then aligned to the assembled contigs to assess the integrity and quality of the assembly. The concordant paired mapped alignment rate was 91.47%. Furthermore, the single-copy homologous gene pool used to assess genetic spatial integrity showed a BUSCO [35] of 97.9% for the assembled genome, highlighting its good completeness.

### Scaffold construction and curation

Hi-C is a high-throughput chromosome conformation capture technology. Taking the entire nucleus as the research object, this approach fixes and captures the interacting parts of the chromosome, followed by high-throughput sequencing to evaluate the spatial distribution of chromatin DNA throughout the genome and to obtain high-resolution chromosomal regulatory elements from positional relationships [36, 37]. In this study, we used 50 Gb of Hi-C data (100× coverage) to generate chromosome-level super-scaffolds. Subsequent analysis of the Hi-C library results showed that the genome was 423 Mbp with a scaffold N50 of 55 Mb. Compared with those of previous red clover sequence data (scaffold N50 = 223 kb), the quality and integrity were substantially better. After Hi-C-assisted assembly, 412 Mbp of genome sequence was mapped to seven chromosomes, accounting for 97.32% of the total sequence. Inter- and intra-chromosomal linkages were calculated to further verify the accuracy of the assembly. The linkages within chromosomes were much stronger than those between chromosomes, as revealed by the Hi-C heatmap. Furthermore, interactions were stronger for chromosomes at closer physical locations than at more distant physical locations (Fig. 1b). These results demonstrate the high accuracy of the assembled genome. Table 1 summarizes the assembly information.

**Table 1:** Summary statistic for the *Trifolium pratense* genome

	Assembly	
Genome assembly	Estimated genome size	456Mbp
	Total length of assembly	423Mbp
	Number of contigs	194
	Contig N50	13Mbp
	Largest contig	34Mbp
	Number of scaffolds	96
	Scaffold N50	55Mbp
	Chromosome coverage (%)	97.32%
	GC content of genome	33.6%
	Annotation	
		Total length
Transposable elements	Total	224Mbp (52.81%)
	Retrotransposon	140Mbp (33.04%)
	DNA Transposon	37Mbp (8.69%)
		Copies
Noncoding RNAs	rRNAs	13,053
	tRNAs	1281
	miRNAs	477
	snRNAs	990
Gene models	Number of genes	44,588
	Mean gene length	3620 bp
	Mean coding sequence length	1585 bp

### Genome annotation

Gene functions in the genome are inferred by computational homology-based alignments and the prediction of repeat sequences. In this study, we identified miniature inverted-repeat transposable elements (MITEs) and long terminal repeat (LTR) transposable elements. Structural features were used for prediction, and these elements accounted for 52.81 and 30.01% of the total sequences, respectively. LTR-retrotransposons classified as Copia and Gypsy accounted for 11.26 and 7.70%, respectively, and 7605 simple repeats were found in the assembled genome. In addition, 16,227 ncRNAs were found and included 13 types.

Similarly, 44,588 high-confidence gene models and 46,089 transcripts were obtained using RNA-seq and de novo prediction strategies after removing the gene models containing early stop codons and frameshifts [38]. The gene models were unevenly distributed across the seven chromosomes.

**Fig. 2:**
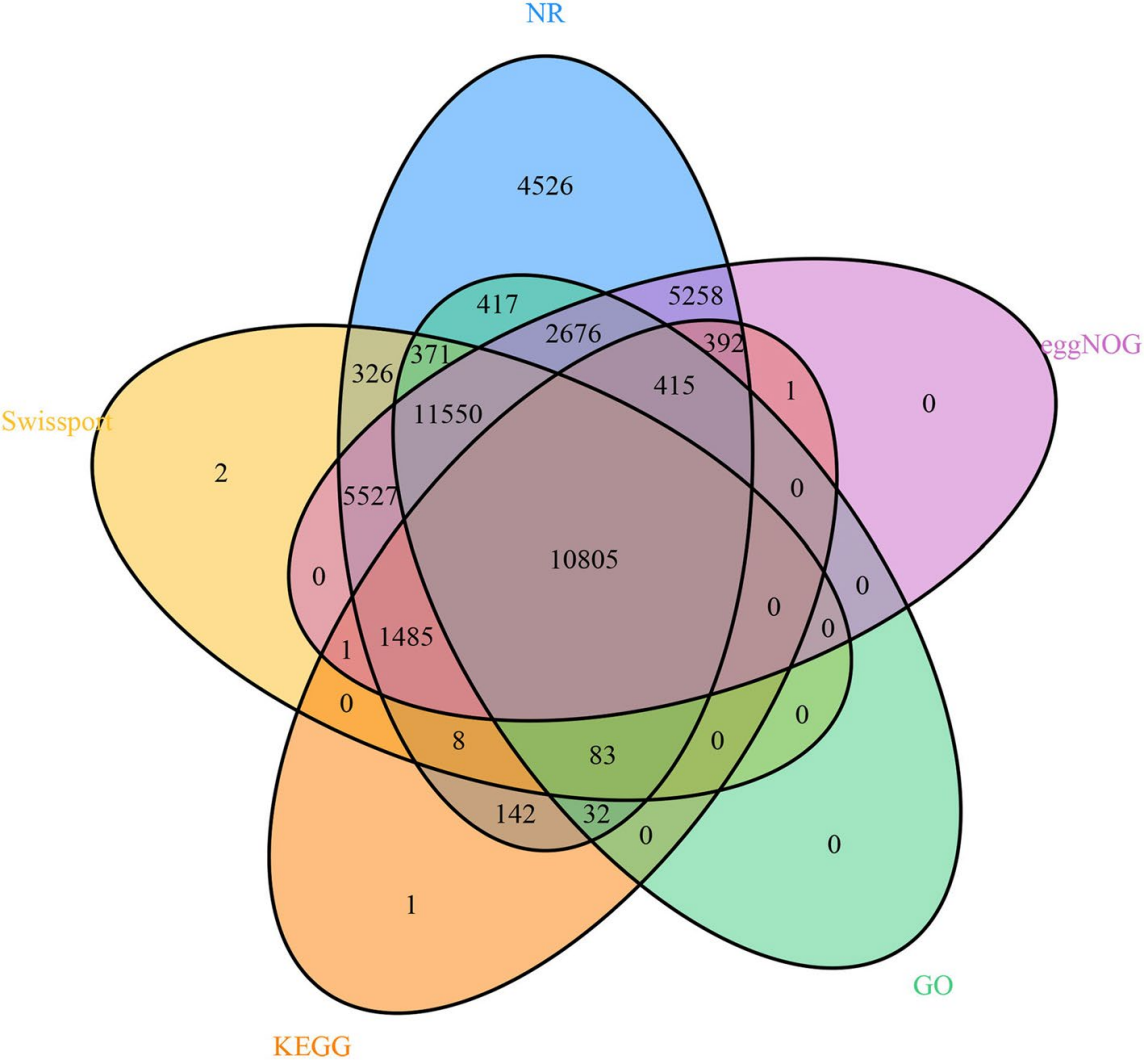
Venn analysis of five major databases (NR, Swiss-Prot, eggNOG, GO, KEGG) containing gene function annotation information

The average lengths of genes and transcripts were 3620 bp and 1730 bp, respectively. Each gene contained an average of 1 transcript, and each transcript contained an average of 5 exons. The average lengths of CDS, exons, and introns were 1585 bp, 348 bp, and 495 bp, respectively. In addition, we compared the genome with those of five closely related species, including *Medicago truncatula* (MtrunA17r5.0-ANR from NCBI), *Trifolium medium* (ASM349008v1 from NCBI), *Vigna radiate* (ver6 from NCBI), *Cicer arietinum* (ASM33114v1 from NCBI)*,* and *Glycine max* (v4.0 from NCBI). Among these, soybean had the largest genome (1012 Mb) [39], which was 2.4 times larger than the red clover genome. The *T. meduium* assembly had the largest number of genes (119102), *C. arietinum* the fewest (28772), and the other three closely related species had similar gene counts and average CDS lengths (Table 2). Using the NR, SwissProt, KEGG, GO, and eggNOG databases, 44,018, 30,158, 13,365, 26,349, and 38,110 genes were annotated, and gene functions and counts were obtained. A dataset of 10,805 common genes was visualized by a Venn diagram (Fig. 2, Table S1).

**Table 2:** The information of annotated gene models per species for all the species

Organism	Number of genes	Mean CDS length (bp)	Exons per transcript	Mean exon length (bp)	Mean intro length (bp)
*Vigna radiata*	29,006	1430	7.6	293	449
*Glycine max*	54,881	1391	8	295	413
*Trifolium medium*	119,102	306	1.4	219	172
*Cicer arietinum*	28,772	1393	7.7	291	418
*Medicago truncatula*	36,079	1428	6.9	324	393
*Trifolium pratense*	44,588	1585	5	348	440

### Gene family and evolution analysis

**Fig. 3:**
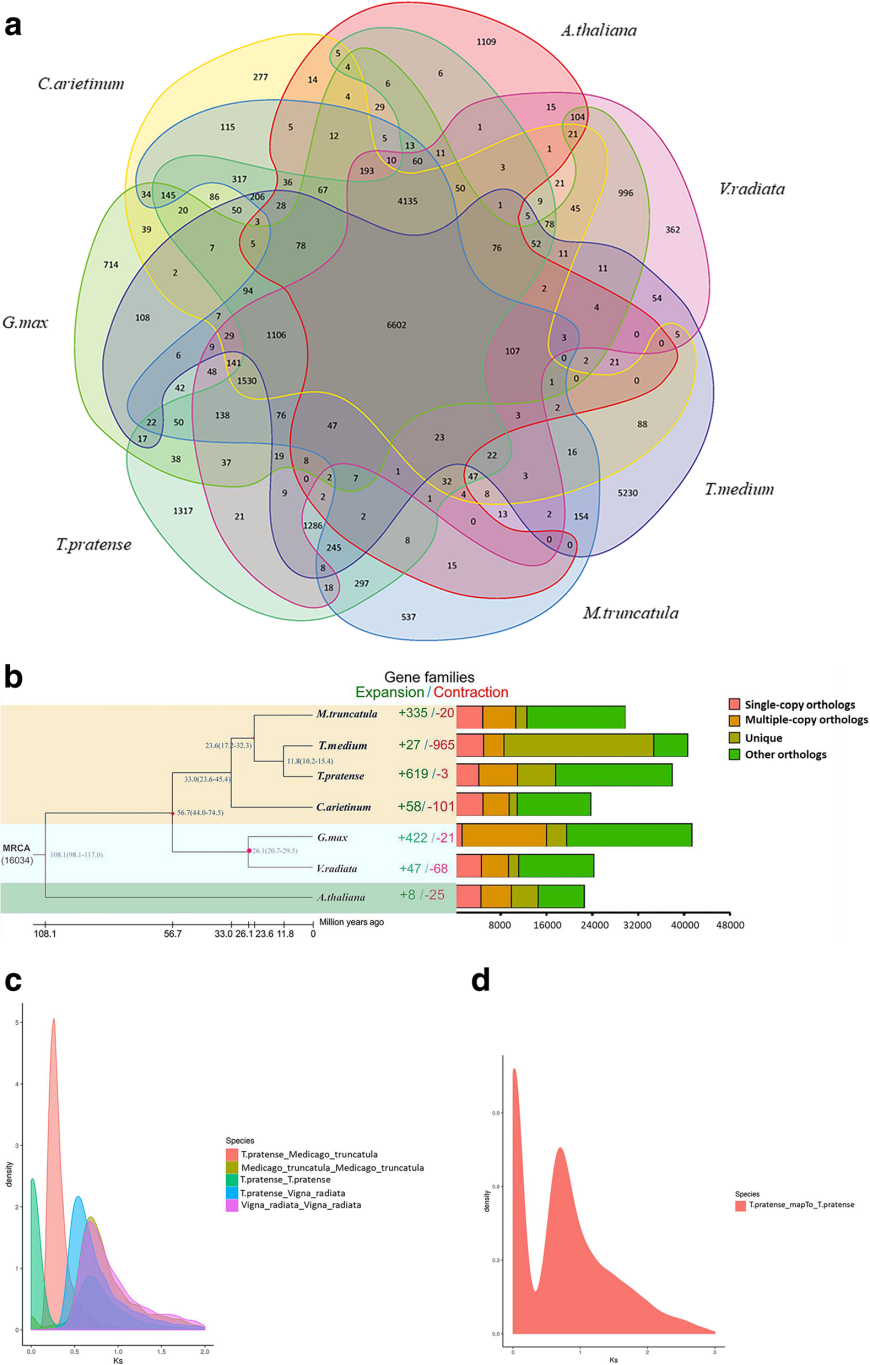
Gene family and phylogenetic tree analyses of red clover and other representative plant genomes. **a** Venn diagram of the number of shared gene families. **b** A phylogenetic tree based on shared single-copy gene families (left), gene family expansions and contractions among red clover and seven other species (middle), and Gene family clustering in red clover and seven other plant genomes (right). **c** Genome-wide replication Ks distribution map of red clover and its related species. **d** Genome-wide replication Ks analysis of red clover

Comparing the genome of red clover with those of six representative species, a gene family analysis revealed that 44,588 genes clustered in 29,508 gene families. Among species, *T. medium* had the most gene families (5230). As visualized by a Venn diagram, 6602 gene families were shared by all species (Fig. 3a). Furthermore, over 11.8 million years of differentiation between red clover and *T. medium*, 619 gene families in red clover expanded and 3 gene families contracted (Fig. 3b). A GO enrichment analysis showed that the expanded gene families were enriched in terms such as ADP binding, oxidoreductase activity and defense response (Table S2). A GO enrichment analysis of genes with the signature of positive selection, as detected by the branch-site test, was also performed [40]. Most of the genes in red clover under positive selection were related to biosynthetic reactions (Table S3). We speculate that the expanded gene families and genes under positive selection may be involved in stress resistance and energy metabolism, which could help to increase the environmental adaptability of plants.

**Fig. 4:**
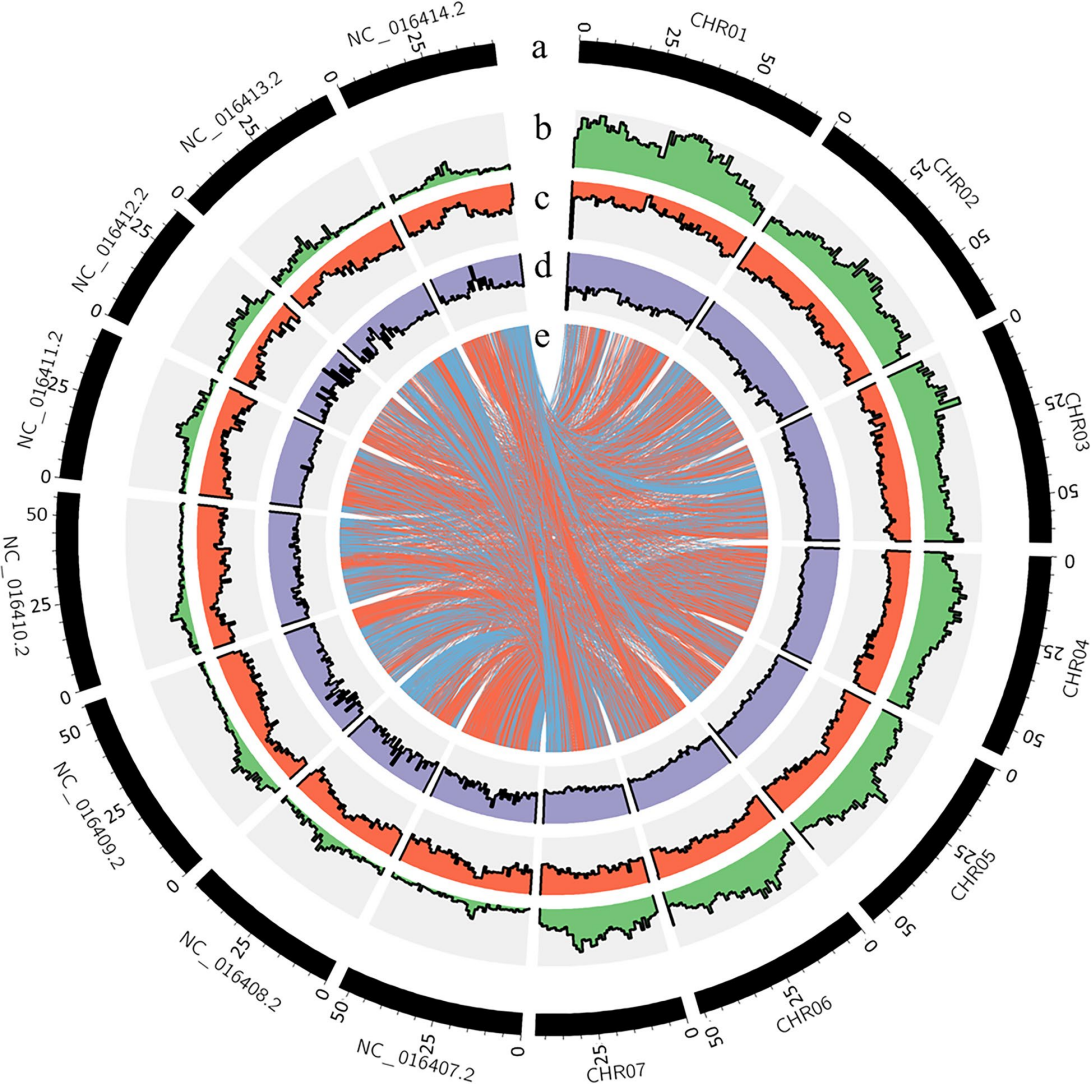
Features of the red clover and *M.truncatula* genome. **a** Length of each pseudochromosome (Mb). **b** Distribution of repetitive sequence. **c** Distribution of gene density. **d** Distribution of the GC content (**e**) Red clover and *M.truncatula* synteny analysis; the beginning of NC represents the chromosome of *M.truncatula,* while the beginning of CHR represents the chromosome of red clover

A phylogenetic tree was constructed based on 754 single-copy homologous genes, with *Arabidopsis* (TAIR10.1 from NCBI) as the outgroup [41]. Red clover clustered with *G. max*, *V. radiata*, *M. truncatula*, *T. medium,* and *C. arietinum* to form a monophyletic group. Red clover was most closely related to *T. medium,* with an estimated divergence time of about 12 million years ago. The collinearity of several closely related species was then evaluated, revealing that red clover and *M. truncatula* show a certain degree of synteny (Fig. S1). The seven chromosomes of red clover and eight chromosomes of *M. truncatula* had good collinearity (Fig. 4), indicating high conservation after species divergence.

Whole genome duplication (WGD) events are important indicators of plant evolution and are considered to be the driving force behind plant adaptation to various environments [42]. Using differences in synonymous substitution rates to detect gene duplication and loss in the red clover genome. The results showed that the divergence of red clover occurred prior to the WGD event shared by *M. truncatula* and *V. radiata*. Red clover experienced a common WGD event with *M. truncatula* and *V. radiata* with *K*_S_ values of 0.28 and 0.67, respectively (Fig. 3c). In addition, a WGD event in red clover also corresponded to a *K*_S_ peak of 0.75 (Fig. 3d).

## Discussion

Genetic and genomic data for leguminous plants with excellent agronomic traits are important resources for the study of genetics, breeding, and functional omics. In this study, we assembled a high-quality diploid genome for a widely important forage crop, the autodiploid red clover. A highly contiguous and complete reference genome is necessary for detailed population genetics analyses and experimental studies. The new genomic data contribute to the legume genome resource bank and provide a good reference for further research on other crops in the clover genus. By combining state-of-the-art technologies (including PacBio HiFi, Hi-C, and high-quality genome assemblers), we generated a representative red clover diploid genome. Compared with the previously reported red clover reference genome, the continuity and completeness of the newly assembled red clover genome showed a high quality: 13 Mbp contig N50, 55 Mb scaffold N50, and 97.9% BUSCO score. The assembly result was high-quality in comparison with legume genomes published to date. Notably, after combining high-throughput sequencing and Hi-C scaffolding, the total sequence length across all seven chromosomes was 412 Mbp, which was very similar to the size of the assembled genome (423 Mb). Genome annotation revealed 44,588 genes with high confidence models, providing an important resource for molecular breeding and evolutionary studies.

Red clover belongs to the legume family, and its evolutionary history is not well-understood. Our analyses of gene family expansion and contraction and positive selection revealed the molecular basis underlying survival under geographical environment and climate change as well as its excellent agronomic traits. The genetic information provides very important resources for future research on legume improvement and stress-resistant species. According to a phylogenetic analysis, *Trifolium* species arose after the differentiation of *G. max*, *V. radiata*, *M. truncatula,* and *C. arietinum*, followed by the divergence of red clover and *T. medium*. Red clover and *Medicago truncatula* showed good genomic collinearity and were closely related based on the phylogeny and sequence similarity. The genome of red clover will help to clarify the evolutionary processes shaping legume species.

## Conclusion

We report a high-quality chromosomal-level red clover genome assembled using third-generation HiFi sequencing. The newly generated genome has the highest coverage and integrity among published legume genomes. This study provides an excellent genetic resource for molecular breeding and the improvement of legumes and provides an important basis for research on the evolution of legumes.

### Experimental procedures

For genomic DNA sequencing, the leaves samples of well-grown from single red clover were collected by a professional graduate student and were collected into vacutainer tubes. Red clover (2n = 14) was grown in a light incubator at the Grassland Agri-husbandry Research Center. The study followed ethics norms and was in compliance with Chinese and international regulations.

### DNA isolation and sequencing

The red clover Emerson was chosen as the test plant. Plants were grown in an incubator at the Qingdao Agricultural University in Shandong, China. After the leaves were treated with liquid nitrogen, DNA was extracted using the Tiangen DNA Secure Kit for Genome Sequencing (Beijing, China). Genome sequencing was performed by Berry Hekang (Beijing, China) using the third-generation PacBio Sequel II assembly sequencing platform. The extracted DNA was used for library preparation and paired-end (PE) sequencing of the library using Illumina NovaSeq [30, 43, 44]. Sequence reads containing adapters, repeats, and low-quality reads (quality scores < 20) were first filtered out. Then, 10,000 reads were randomly selected for NT comparison using the BLAST tool [45]. No apparent external contamination was detected. A K-mer analysis was used to estimate the genome size, duplication rate, and heterozygosity. 17-mer frequency distribution analysis of quality-filtered reads by Jellyfish [28]. The genome size of red clover was estimated using the following formula: *G* = *K*_num_/*K*_depth_, where *K*_num_ is the number of k-mers, *K*_depth_ is the expected depth of *k*-mers.

The quality of genomic DNA was checked using a NanoDrop 2000 spectrophotometer. The purified genome was used to construct the SMRTbell library and sequenced using PacBio SMRT technology [46, 47]. The library size was determined using an Agilent 2100 Bioanalyzer (Santa Clara, CA, USA). The acquired data were filtered and then processed using smrtlink for CCS processing.

### Genome assembly and quality evaluation

HiFi reads were assembled using hifiasm, and purge-dups was used to remove hybridized fragments in the contig sequence [48, 49]. A single-copy orthologous gene library using a combination of tblastn, augustus, and hummer was finally used to assess the integrity of the assembled genome [35].

### Hi-C data analysis and chromosome construction

The leaf tissue (100 mg) of red clover which was the same individual used in HiFi sequencing. It was soaked in paraformaldehyde, a cell cross-linking agent, for 15 minutes. Glycine was then added to the mixture to stop the chromatin cross-linking reaction, and the treated tissue was collected and frozen in liquid nitrogen. These tissues were then ground into a powder to extract DNA. Biotin-labeled oligonucleotide ends were added during end-repair, and the extracted DNA was subsequently fragmented into 350 bp fragments using a Covaris breaker [36, 50]. The biotin-conjugated DNA was captured and purified using avidin magnetic beads, and the library was constructed and sequenced using the Illumina PE150 platform [37, 51]. The raw reads were filtered, and 10,000 randomly selected sequencing reads were aligned with the NT library using the BLAST tool. The Hi-C data and draft genome were then compared using JUICER [52]. The Hi-C library results were subsequently analyzed using 3D-DNA alignments to obtain valid Hi-C data and generate the chromosome-level scaffold of the red clover genome [53].

### Functional annotation

Repeat sequences were analyzed and predicted using RepeatMasker, MITE Hunter, LTRharvest, LTR Finder, LTR retriever, and RepeatModeler, and MITES and LTR transposable elements were identified using structure-based prediction methods [54–59]. The software parameters for LTRharvest and LTR Finder were -similar 90 -vic 10 -seed 20 -seqids yes -minlenltr 100 -maxlenltr 7000 -mintsd 4 -maxtsd 6 -motif TGCA -motifmis And -D 15000 -d 1000 -L 7000 -l 100 -p 20 -C -M 0.9 [60]. The parameters settings for RepeatModeler for the de novo identification of repeat sequences in masked genomes were -engine ncbi -pa 60. Similarly, the parameters for RepeatMasker for masking repeat sequences in the genome were -s -nolow -norna -gff -engine ncbi -parallel 20 [56]. tRNAscan-SE was used to predict tRNA ab initio rRNA [61]. The Rfam database was used to search for other types of ncRNAs [62, 63]. Specific information was obtained by a similarity analysis.

Except for tandem repeats, all repeat regions were soft-masked and subsequently used for the annotation of protein-coding genes [64]. The coding sequences of *G. max, V. radiata, M. truncatula, T. medium,* and *C. arietinum* were downloaded. These coding sequences were subjected to Blast (v. 2.2.20) searches against the red clover genome [45]. Homologs containing premature stop codons and frameshifts were discarded. Red clover RNA-seq data from different tissues were aligned to red clover contigs using GeMoMa-1.6.1 and a comprehensive transcriptome database was built using PASA (v. 2.0.1) [65, 66]. Open reading frames were predicted using PASA (v. 2.0.1) and the resulting database was used to train parameters for the following four de novo gene prediction software packages: AUGUSTUS (v. 3.2.2) [67], GeneMarker-ET (v. 4.57) [68], GlimmerHMM (v. 3.0.2) [69], and SNAP [70]. Predictions obtained using these packages were then combined using EVM [71, 72], and 44,588 genes were retrieved and functionally annotated by blast searches against databases, including NR, Swiss-Pro, eggNOG, GO, and KEGG [73–75]. A Venn diagram of the genes obtained from the five major databases was then generated to obtain more accurate annotation information.

### Comparative analysis

A collinearity analysis of red clover and closely related species was performed using MCscan with parameters -g 1000 -c 90 -l 200 [76]. Notably, the OrthoMCL cluster analysis was used to identify seven gene protein families (Red clover, *G. max*, *V. radiata*, *M. truncatula*, *T. medium*, *A. thaliana,* and *C. arietinum*) [77]. First, an all-vs.-all BLAST alignment of the protein-coding sequences of all of the above species was generated, and the similarity between the sequences was calculated [78]. The Markov clustering algorithm (expansion coefficient of 1.5) was used for protein family identification [42, 79, 80]. Single-copy genes in each species were selected as reference markers. Four-fold degenerate sites were selected to construct supergenes [81]. Multiple sequence comparisons of supergenes were subsequently performed using Mafft [82, 83]. An appropriate base substitution model was selected and a species-based maximum likelihood (ML) phylogenetic tree was constructed using RAxML [84–86]. The mcmctree tool in the PAML software package was used to estimate the differentiation times based on 754 single-copy homologous genes [87, 88]. The single-copy homologous genes were identified by OrthoMCL, a correlated molecular clock model and a REV substitution model [89]. After a burn-in of 5,000,000 iterations, the MCMC process was repeated 1,000,000 times with a sample frequency of 50. The times were further calibrated using the predicted divergence times of *M. truncatula-T. pratense* (17.23–32.3 Mya), *M. truncatula- V. radiata* (44.0–74.5 Mya) and *G. max-V. radiata* (20.7–29.5 Mya) based on available TimeTree (http://timetree.org) [90]. Then, the variation in gene family sizes among species was analyzed using Café, followed by a GO functional enrichment analysis of the expanded gene families [91]. The branch-site model was used to detect positive selection on specific branches and affecting a portion of sites by selecting one-to-one orthologous proteins from red clover and its closely related species and using PRANK [40]. After aligning homologous protein sequences, Gblocks was used to filter the alignment results [92]. CODEML in PAML was used to evaluate positive selection in specific clades and affecting only certain loci with correction for multiple hypothesis testing using Chi2 [88]. The duplicate age distribution was used to detect WGD events, and blastp was used to align the longest protein sequences for genes in the red clover genome [93]. The results were then filtered using MCScanX and synonymous substitution rates were calculated using the Yn00 tool in the PAML software package [94]. A density distribution map based on the *K*_s_ values for all paralogous gene pairs and *K*_s_ values of ortholog gene pairs between the genomes of red clover, *M. truncatula,* and *V. radiata* were then drawn using Matlab [95].

## Supplementary Information

**Additional file 1: Table S1.** GO, eggNOG, NR, KEGG and SP annotation results.

**Additional file 2: Table S2.** The gene families described (including their GO terms) and their numbers between red clover and the gene family expansion in red clover.

**Additional file 3: Table S3.** The genes in red clover under positive selection were described (including their GO terms) and their numbers.

**Additional file 4: Table S4.** URLs and code of the software.

**Additional file 5: Figure S1.** Red clover and its closely related species synteny analysis. (**a**) The beginning of NC represents the chromosome of *M.truncatula,* while the beginning of CHR represents the chromosome of red clover. (**b**) The beginning of NC represents the chromosome of *C.arietinum,* while the beginning of CHR represents the chromosome of red clover. (**c**) The beginning of NC represents the chromosome of *G.max,* while the beginning of CHR represents the chromosome of red clover. (**d**) The beginning of NC represents the chromosome of *V.radiata,* while the beginning of CHR represents the chromosome of red clover.

## Data Availability

All data generated and analyzed during this current study are available in the Grassland Agri-husbandry Research Center, Qingdao Agricultural University with permission from the Competent Authority. All sequencing data were submitted in NCBI Database having BioProject ID PRJNA765108 and details of software used are in Table S4. Biological materials used in this study available from the corresponding author.

## Abbreviations

NT: Nucleotide Sequence Database
NGS: Next-Generation Sequencing
CCS: Circular Consensus Sequencing
BUSCO: Benchmarking Universal Single-Copy Orthologs
Hi-C: High-through chromosome conformation capture
MITEs: miniature inverted repeat transposable elements
LTR: Long terminal repeat
LTR-RT: Long terminal repeat retrotransposons
ncRNA: Non-coding RNA
NR: NCBI nucleotide sequences
GO: Gene Ontology
KEGG: Kyoto Encyclopedia of Genes and Genomes
WGD: Whole genome duplications
