# Supplementary material for: A de novo assembled high-quality chromosome-scale Trifolium pratense genome and fine-scale phylogenetic analysis
Source: BMC Plant Biol. 2022 Jul 11;22:332. doi: 10.1186/s12870-022-03707-5 (PMC9277957; doi:10.1186/s12870-022-03707-5)
Supplement: Supplementary file 5 — Additional file 5: Figure S1. Red clover and its closely related species synteny analysis. (a) The beginning of NC represents the chromosome of M.truncatula, while the beginning of CHR represents the chromosome of red clover. (b) The beginning of NC represents the chromosome of C.arietinum, while the beginning of CHR represents the chromosome of red clover. (c) The beginning of NC represents the chromosome of G.max, while the beginning of CHR represents the chromosome of red clover. (d) The beginning of NC represents the chromosome of V.radiata, while the beginning of CHR represents the chromosome of red clover. [file 12870_2022_3707_MOESM5_ESM.zip › Additional file 5ú║Figure S1 and legend/Legend of figure S1.pdf]

Fig. S1 Red clover and its closely related species synteny analysis. (**a**) The beginning of NC represents the chromosome of *M.truncatula,* while the beginning of CHR represents the chromosome of red clover. (**b**) The beginning of NC represents the chromosome of *C.arietinum,* while the beginning of CHR represents the chromosome of red clover. (**c**) The beginning of NC represents the chromosome of *G.max,* while the beginning of CHR represents the chromosome of red clover. (**d**) The beginning of NC represents the chromosome of *V.radiata,* while the beginning of CHR represents the chromosome of red clover.
